# Book Reviews

**Published:** 1901-04

**Authors:** 


### BOOK REVIEWS, PAMPHLETS, EXCHANGES.

### BOOK REVIEWS.

[Contributions solicited for review.]

A System of Practical Therapeutics. By eminent American and Foreign Authorities. Edited by Hobart Amory Hare, M.D., Professor of Therapeutics, Jefferson Medical College; Physician to Jefferson College Hôspital, etc., Philadelphia. New (2d) edition, thoroughly revised. In three very handsome octavo volumes, containing 2593 pages, with 427 engravings, and 26 full-page colored plates. Per volume, cloth, $5.00, net; leather, $6.00, net; half morocco, $7.00, net. Lea Brothers & Co., Publishers, Philadelphia and New York, 1901.

The second volume of this system contains able practical sections on the following subjects: Typhoid Fever (new), by H. A. Hare, M.D., Philadelphia; Malarial Fevers, by James M. Anders, M.D., LL.D., Philadelphia; Smallpox, by William M. Welsh, M.D., Philadelphia; Varicella, Rubeola, Rubella and Scarlatina (new), by J. P. Crozer Griffith, M.D., Philadelphia; Yellow Fever (new), by D. T. Lainé, M.D., Havana; Dengue (new), by J. W. McLaughlin, M.D., Galveston; Acute Tonsillitis and Influenza, Acute Articular Rheumatism (new), by Frederick A. Packard, M.D., Philadelphia; Diphtheria (new), by Floyd M. Crandall, M.D., New York; Spasmodic Croup and Rickets (new), by Floyd M. Crandall, M.D., New York; Diseases of the Mucous Membrane of the Mouth, and Mumps (new), by Floyd M. Crandall, M.D., New York; Pneumonia, Croupous and Catarrhal (new), by H. A. Hare, M.D., Philadelphia; Asthma, Bronchitis and Whooping-cough, by Norman Bridge, M.D., Chicago; Acute and Chronic Organic Diseases of the Heart, by W. H. Thomson, M.D., LL.D., New York; Diseases of the Blood-Vessels, by Frederick C. Shattuck, M.D., Boston; Nervous Diseases of the Heart, by Sir Lauder Brunton, M.D., D.Sc., LL.D. Edin., LL.D. Aberd., F.R.C.P., F.R.S., London; Diseases of the Stomach, by Thomas G. Ashton, M.D., Philadelphia; Diseases of the Liver, Gall-bladder, Hepatic Duct, and Spleen (new), by John H. Musser, M.D., Philadelphia; Diarrhœal Diseases and Dysentery, by W. W. Johnston, M.D., Washington; The Intestinal Parasites, by H. A. Hare, M.D., Philadelphia; Diseases of the Kidneys, by N. S. Davis, Jr., M.D., Chicago; Headaches and Neuralgia, by Wharton Sinkler, M.D., Philadelphia; The Drug-Habits, by F. X. Dercum, M.D., Philadelphia; The Disorders of Sleep, by Hugh T. Patrick, M.D., Chicago; Locomotor Ataxia, Acute Infantile Spinal Paralysis, Myelitis, and Amyotrophic Lateral Sclerosis, by M. Allen Starr, M.D., Ph.D., New York; Apoplexy, Brain Tumor, Spinal Tumor, Meningitis, Cerebritis, and Neuritis, by Charles K. Mills, M.D., Philadelphia; Spasmodic Affections of the Nervous System, by Joseph Collins, M.D., New York; The Medical Treatment of Insanity, by H. M. Bannister, M.D., Kankakee, Ill.; Hospital Treatment of Insanity, by Edward N. Brush, M.D., Baltimore; The Modern Treatment of Diseases of the Skin, by Henry W. Stelwagon, M.D., Philadelphia.

---

DISEASES OF THE STOMACH. Their Special Pathology, Diagnosis, and Treatment, with Sections on Anatomy, Physiology, Chemical and Microscopical Examination of Stomach Contents. Dietetics, Surgery of the Stomach, etc. By John C. Hemmeter, M.D., Ph.D., Professor in the Medical Department of the University of Maryland, Baltimore; Consultant to the University Hospital, and Director of the Clinical Laboratory, etc., with many Original Illustrations, a number of which are in Colors, and a Lithograph Frontispiece. Second enlarged edition, with new chapter and additional illustrations. Published by P. Blakiston's Son & Co., 1012 Walnut St.. Philadelphia, 1900. Price, $6.00, net.

The author has so thoroughly and critically revised this edition of his classical work, that it has practically been reconstructed. There being less than fifty pages that have not received alteration, or some important addition, shows the great amount of energy and time expended in bringing the subject-matter fully up to the present status of our knowledge of this important branch of Medical Science. The book is handsomely and profusely illustrated throughout. The original illustrations and plates are especially good, but all of them serve to elucidate the text most admirably. We know of no book on the subject more worthy of a hearty reception at the hands of the medical profession, and that it will have a large sale there can be no doubt. The publishers are to be commended for their excellent press work.

---

STUDENTS' EDITION, A PRACTICAL TREATISE OF MATERIA MEDICA AND THERAPEUTICS, with special reference to the Clinical Application of Drugs. By John V. Shoemaker, M.D., LL.D., Professor of Materia Medica, Pharmacology, Therapeutics, and Clinical Medicine, and Clinical Professor of Diseases of the Skin in the Medico-Chirurgical College of Philadelphia; Physician to the Medico-Chirurgical Hospital; Member of the American Medical Association of the Pennsylvania and Minnesota State Medical Societies, the American Academy of Medicine, the British Medical Association; Fellow of the Medical Society of London, etc., etc. Fifth edition. Thoroughly revised. 6¼x 9½ inches. Pages vii-770. Extra Cloth, $4.00, net; Sheep, $4.75, net. F. A. Davis Company, Publishers, 1914-16 Cherry Street, Philadelphia.

It was deemed expedient to the author and publishers to divide this work into an edition for students and one for practitioners not yet published. The students' edition contains only an account of the official drugs, poisons and their antidotes, pharmacy and prescription writing and general therapeutic classification of remedies. This plan will be especially acceptable to students and will commend itself to the teacher of this branch. Shoemaker's work has long been popular and this edition is in no way behind the others.

---

THE TREATMENT OF FRACTURES. By Chas. L. Scudder, M.D., Assistant in Clinical and Operative Surgery, Harvard Medical School. Second edition, revised and enlarged. Octavo, 433 pages, with nearly 600 original illustrations. Philadelphia and London: W. B. Saunders & Co., 1901. Polished Buckram, $4.50 net.

This book is intended to serve as a guide to the practitioner and student in the treatment of fractures of bones, being a practical statement of the generally recognized methods of dealing with fractures. The attention of the student is diverted from theories to the actual conditions that exist in fractured bones, and he is encouraged to determine for himself how to meet the conditions found in each individual case. Methods of treatment are described in minute detail, and the reader is not only told, but is *shown*, how to apply apparatus, for, as far as possible, all the details are illustrated. This elaborate and complete series of illustrations constitutes a feature of the book. There are nearly 600 of them, all from new and original drawings and reproduced in the highest style of art.

The exhaustion in a remarkably short time of a large first edition indicates the favor with which this work has been received. From the day of its issue it has been accorded the highest possible praise, as being the most practical and useful work on the treatment of fractures that has ever been issued. In this edition the book has been thoroughly revised. Many X-ray plates have been reproduced to assist in familiarizing the reader with the study of such plates. Physicians should be able to interpret X-ray plates without the assistance of an expert. Numerous other new illustrations have been added, and the book has been considerably enlarged.

---

DISEASES OF THE NOSE AND THROAT. By D. Braden Kyle, M.D., Clinical Professor of Laryngology and Rhinology, Jefferson Medical College, Philadelphia; Consulting Laryngologist, Rhinologist, and Otologist, St. Agnes' Hospital. Second edition, revised. Octavo, 646 pages; over 150 illustrations and 6 lithographic plates. Philadelphia and London: W. B. Saunders & Co., 1901. Cloth, $4.00, net.

The first edition of this work was exhausted in less than a year. The author has accordingly taken the opportunity of thoroughly revising the book and bringing the subject-matter absolutely down to date. The work presents the subject of Diseases of the Nose and Throat in as concise a manner as is consistent with clearness, keeping in mind the needs of the student and general practitioner as well as those of the specialist. The illustrations are particularly fine, being chiefly original. With the practical purpose of the book in mind, extended consideration has been given to details of treatment, each disease being considered in full, and definite courses being laid down to meet special conditions and symptoms.

PULMONARY CONSUMPTION, PNEUMONIA AND ALLIED DISEASES OF THE LUNGS. Their Etiology, Pathology and Treatment, with a chapter on Physical Diagnosis. By Thomas J. Mays, A.M., M.D., Professor Diseases of the Chest in the Philadelphia Polyclinic; Visiting Physician to Rush Hospital for Consumption. Illustrated. New York: E. B. Treat & Co., 241, 243 W. 23d St., 1901. Price, $3.60.

In this book the author sets forth at great length his novel beliefs as to the cause and treatment of pulmonary consumption and allied diseases of the lungs. These beliefs he has formulated into the following propositions:

1. That pulmonary phthisis in the large majority of cases is primarily a neurosis, and that the pulmonary disintegration is secondary.
2. That any agent or influence which undermines the integrity of the nervous system will engender pulmonary phthisis or some other form of pulmonary disorder.
3. That the only remedies of value in the treatment of pulmonary phthisis are those which appeal to and act through the nervous system.
4. That of special value in the treatment of phthisis is the counterirritant action of nitrate of silver introduced hypodermically over the vagi in the neck; and
5. That acute pneumonia and other forms of acute pulmonary disease are closely affiliated with disorder of the nervous system."

Of these doctrines it may be said that either they are retrogressive or that they are so far in advance of the times as to be beyond the horizon. A possible good that might accrue from them is in an amplification of the scope of the neurologist.

---

DISEASES OF CHILDREN. Taylor & Wells. Second edition. Illustrated. Price $4.50. P. Blakiston's Son & Co., 1012 Walnut St., Philadelphia, 1901.

The second edition of this excellent work shows many new features and improvements over the first. New chapters have been added and special articles revised and remodeled, making the present volume much larger than the former one. The work is comprehensive and very well covers the whole field of pediatrics. It is exceedingly well printed and handsomely gotten up. Those who are in need of a new and reliable book on Diseases of Children will not be disappointed in this.

HYPNOTISM. A Complete System of Method, Application and Use, prepared for the self-instruction of the Medical Profession. By L. W. DeLaurence, instructor at the School of Hypnotism and Suggestive Therapeutics, Pittsburg. Chicago: The Henneberry Co. Publisher's price $1.50.

Those interested in the subject of hypnotism and suggestive therapeutics will find in this book a fairly thorough exposition of the subject. The author has presented every phase of the subject, and the medical practitioner would do well to gain a knowledge of this therapeutic resource, to be able to avail himself of its benefit in selected cases.

---

MANUAL OF VENEREAL DISEASES. Sturgis and Cabot. Seventh edition. Price $1.25 net. P. Blakiston's Son & Co., Philadelphia, 1901.

This manual is intended for students of medicine, and deals concisely with the commoner forms of venereal disease likely to fall under the notice of the young practitioner. It is written from the point of view of a lecturer addressing himself to his class, and has, therefore, the force and simplicity of a clinical exposition. The student may gain from the book a sound working knowledge of venereal disease, and the practitioner will find profit in reading it.

---

PROGRESSIVE MEDICINE. Vol. I. March, 1901. Lea Bros. & Co., Philadelphia and New York.

The current volume is concerned with the Surgery of the Head, Neck and Chest, Infectious Diseases, including Acute Rheumatism, Croupous Pneumonia and Influenza, Diseases of Children, Pathology, Laryngology and Rhinology, Otology. The contributors are Floyd M. Crandall, J. C. Da Costa, Hektorn, Packard, Randolph, Turner. The volume is in the usual excellent form of the publishers, and gives the progress of medicine in these sections up to the date of issue.

---

INTERNATIONAL CLINICS. Vol. IV. Tenth Series. J. B. Lippincott Co., Philadelphia, 1901.

The notable contributions to this volume are a finely illustrated article on Mosquitoes and the Prophylaxis of Malaria, by Prof. B. Grassi, one of the most noted investigators on this subject; a symposium of genito-urinary diseases, including articles by Fournier & Guyon. Besides these there are the usual additional lectures covering a wide field of interest. The book is, as usual, well illustrated and handsomely bound.

